# Male breast cancer: a review

**DOI:** 10.3332/ecancer.2009.140

**Published:** 2009-03-20

**Authors:** IS Fentiman

**Affiliations:** Surgical Oncology, GKT School of Medicine, Guy’s Hospital, London SE1 9RT, UK

## Abstract

Male breast cancer (MBC) is rare, with the peak age of onset at 71 years. BRCA2 mutations are more frequent than BRCA1 with 20% of cases giving a family history. Risk factors for MBC are poorly understood and include working in high-ambient temperatures and exhaust fume exposure. MBC is associated with hyperoestrogenic states found in liver disease, Klinefelter’s syndrome, gonadal dysfunction or obesity. Most information on treatment of MBC is derived from large randomized trials carried out in female patients. The small numbers of MBC seen in any unit annually has precluded significant trials being carried out.

Diagnosis and treatment of MBC is similar to that of female patients, but men tend to be treated with mastectomy rather than breast-conserving surgery. The mainstay of adjuvant therapy or palliative treatment for advanced disease is endocrine, mostly tamoxifen. Prognosis of male patients is equal to that of stage-matched women, but men tend to fare worse because of delay in presentation, leading to a large proportion of patients presenting with stage III or IV disease. Increased input is needed for psychological support for male breast cancer patients. Specific therapeutic questions about MBC need international trials to obtain meaningful answers.

## Background

Male breast cancer is a relatively rare disease in the western world accounting for <1% of all breast cancers. The causes are poorly understood and most studies are small and underpowered so that treatment decisions derive from large trials in post-menopausal women. Although the outlook for men is the same as stage-matched women, overall the prognosis is worse because of delayed presentation—on average 6–10 months from symptom onset to diagnosis [1], with only slight improvement in survival over the last 25 years [2].

The incidence of MBC in Northwest Europe and North America is approximately 1/100,000 but is increasing [3]. Disease frequency is higher among Jewish men at 2.3/100,000 and in countries with a high incidence of parasitic liver disease such as Egypt and Zambia [4,5]. In Japan, where the incidence of female breast cancer is low, the rate of MBC is <0.5/100,000 [6].

## Risk factors

Although several risk factors for MBC have been identified, most men with the disease have none other than increasing age (average age of diagnosis is 71 years, approximately ten years older than in women [7]. Most risk factors relate to either testicular malfunction or an increase in oestrogen (Table 1).

### Genetic

As in women, risk of breast cancer increases with number of first degree relatives with the disease particularly with early age at diagnosis. In 15–20% of MBC cases there is a positive family history [8]. More rarely, men are found to have BRCA1 and BRCA2 mutations, usually the latter. A male BRCA2 carrier has a 6% lifetime risk of developing the disease, compared with 0.1% in the normal population [9]. MBC has been reported in those with Cowden syndrome [10], but not in men with Li Fraumeni syndrome. Individuals with Klinefelter’s syndrome (XXY) have a 20 to 50-fold increased risk of breast cancer and a mortality rate similar to that of females [11,12].

### Occupation

Men working in hot environments such as blast furnaces, steel works and rolling mills have increased risk of MBC [8]. Associations have also been found with occupations involving working with soap, perfume, petrol or exhaust fumes [13,14]. The responsible carcinogens are probably polycyclic aromatic hydrocarbons (PAH), present in tobacco smoke and exhaust emissions. Exposure to electromagnetic fields has been postulated as a risk factor but evidence for this is limited and negative [15].

### Radiation exposure

Radiation exposure increases risk of breast cancer for both women and men [16]; small numbers of chest X-rays do not, but prolonged exposure to radiographs or radiotherapy may be harmful [17]. Radiotherapy has been used in high doses to treat gynaecomastia and a sevenfold increase in the relative risk of breast cancer has been reported in these patients [18]. Some institutions still use low-dose radiotherapy for this condition and the long-term effects of this remain to be seen [19]. Among the 45,880 survivors of an atomic bomb, MBC risk increased up to eightfold, depending on the extent of exposure [20].

### Endocrine factors

MBC risk is affected by oestrogen/testosterone balance with increased rates in men taking exogenous oestrogens, such as prostate cancer patients and transsexuals [21,22]. Testicular dysfunction as a result of congenital inguinal hernia, infertility, testicular injury, orchidectomy and mumps orchitis at age increases risk of MBC by up to twelve-fold [23].

Obesity commonly causes hyperestrogenism in men and some studies suggest that it can double the risk of MBC [24,25]. Liver disease such as cirrhosis also causes hyperestrogenism associated with an increased risk of MBC [26]. A European multi-centre study with 74 cases and 1432 population controls reported a significant relationship between alcohol consumption and risk of MBC [27]: the odds ratio for alcohol intake >90 g/d was 5.89 (CI 2.21–15.69). The risk of MBC rose by 16% for every 10 g of daily alcohol intake. Male breast cancer has been described in patients with hyperprolactinaemia due to pituitary adenomas [28]. There is however no proven link between gynaecomastia and male breast cancer [29].

## Presentation

As in female patients, most males (75%) complain of a painless mass [30]. Other presenting features include nipple retraction, nipple discharge, ulceration and pain [31]. Differential diagnosis includes gynaecomastia, abscess, metastatic disease or rarely, sarcoma. Because the male breast is rudimentary and most tumours are central, nipple involvement is seen in up to 50% of cases at presentation [32]. Despite some increased awareness of MBC, there is still a delay of 6–10 months from onset of symptoms to diagnosis [2]. Of the patients, >40% of men present with stage III or IV disease (Table 2) [29,30,33,34].

## Diagnosis

As with symptomatic female patients, the diagnosis is made by triple assessment, with both mammography and ultrasound scanning having high sensitivity and specificity for male patients [35]. Fine needle aspiration cytology and core biopsies may be used, but core biopsy is preferred because it enables a definitive diagnosis to be made in the majority of cases. MR imaging has little role for determining extent of disease in male patients since the majority will be treated by mastectomy.

## Histopathology

Most male breast cancers are invasive at presentation with only 10% having ductal carcinoma *in situ* (DCIS) (Table 3) [29,32,33,36–38]. The majority are invasive ductal carcinomas (80%) with only 1% of male breast cancers being lobular in origin, since in the male breast, lobule development is dependant upon oestrogenic stimulation.

Paget’s disease and inflammatory breast cancer occur in similar proportions in men and women with breast cancer. Rarer histological subtypes, medullary, tubular, mucinous and squamous have all been reported. Non-invasive disease is usually DCIS of low or intermediate grade, but lobular carcinoma *in situ* (LCIS) has been described, usually coexisting with invasive lobular carcinoma [39].

Oestrogen receptors are expressed in 90% of MBCs, a higher proportion than in women and up to 96% are progesterone receptor positive [40,41]. Human epidermal growth factor receptor 2 (Her-2) overexpression has been reported in 16%, on average [40,42,43] slightly lower than in females but its effects on prognosis are unclear [44].

## Management

The principles of treatment of MBC are similar to those used for females with breast cancer. Men are less likely to have breast-conserving surgery, radiotherapy or receive chemotherapy compared with stage-matched female cases [45]. Most breast units see few MBC patients each decade. Psychological support is very much orientated towards women, but psychological morbidity is also frequent amongst male patients who often have concerns about body image [46]. Additionally, MBC can be a very isolating diagnosis with some patients questioning their masculinity, and these concerns need to be addressed by those who are caring for the patient.

## Treatment of local disease

### Surgery

Surgery is the mainstay of treatment for MBC and mastectomy is the most commonly performed operation because of the size of the male breast and most cancers being central with early nipple involvement. Radical mastectomy was the treatment of choice but this is now infrequently necessary [23]. Tumours close or attached to the pectoralis major can be excised with a small amount of muscle.

Locally advanced tumours can be down staged with neoadjuvant therapy to enable mastectomy. For nonresponders, primary skin closure may not be possible after mastectomy so a latissimus dorsi or rectus sentinel node biopsy (SNB) is the standard of care in men and has the same sensitivity and specificity as that described in female patients [47]. When axillary nodes contain tumour, detected pre-operatively or after SNB a clearance should be performed.

### Endocrine therapy

Since the majority of male breast cancers are oestrogen receptor positive (ER+ve), endocrine therapy is an integral part of treatment. Studies of tamoxifen in men, given for two years, have shown improvement in both disease free, and so the effect of this treatment in men may have been underestimated. Aromatase inhibitors have been used to treat advanced MBC but their adjuvant role has yet to be determined [29].

### Radiotherapy

The few small studies using radiotherapy in men show an improved local control but did not control for other prognostic variables [48,49]. MBC patients should be offered postoperative radiotherapy according to the guidelines drawn up for females. Those few men who have breast-conserving surgery should be offered post-operative radiotherapy. Men with large tumours, locally advanced disease, extensive axillary nodal involvement or poor prognostic factors such as high histological grade and vascular invasion should all be offered post-mastectomy radiotherapy.

### Chemotherapy

Studies of adjuvant chemotherapy in MBC do show a benefit but again these were underpowered [50,51]. Adjuvant chemotherapy for MBC should be advised along the same principles as female patients although age and co-morbidity will render many unsuitable.

## Treatment of advanced disease

Endocrine therapy is the usual treatment for metastatic MBC [52]. Orchidectomy has been used but selective oestrogen receptor modulator (SERM) therapy in the form of tamoxifen is now the treatment of choice. Aromatase inhibitors are not usually used as they only block peripheral oestrogen production that accounts for 80% of oestrogen production in men. There is a risk of aromatase inhibitors up-regulating testicular oestrogen production in those with an intact hypothalamic feedback loop.

Those few men with ER–ve tumours can be treated with chemotherapy. Additionally, the use of androgens, anti-androgens, steroids, oestrogens, progestins and aminoglutethimide for advanced disease in men has also been described.

## Prognosis

Male and female patients with breast cancer are staged similarly, according to AJCC or UICC guidelines. Prognosis depends upon tumour size, histological grade, nodal status and hormone receptor status [53]. The most important prognostic factor is the lymph node status. Those who are node negative can expect a five-year survival rate of 90%, compared with 65% five-year survival rate for node positive cases.

The number of involved nodes is also of prognostic significance: those with 1–3 positive nodes have a ten-year survival rate of 44%, compared with 14% for patients with ≥ four nodes involved [54]. Histological tumour grade also plays a role: grade I patients have a five-year survival rate of 76%, dropping to 66% for those with grade II tumours and 43% for grade III cases. Patients with ER/PR+ve tumours fare better than those with ER/PR–ve disease.

Overall, the prognosis is equivalent to that in stage-matched female patients. MBC patients tend to fare worse because of late presentation associated with more advanced disease. Because MBC cases tend to be older than female patients, there is an increased incidence of co-morbidities, which could adversely affect their prognosis [29].

## Psycho-social aspects

In females with breast cancer, clinically significant anxiety/depression occurs in up to 25% of cases [55,56,57]. Until recently, no structured psychosocial studies have been reported in MBC. In depth interviews of MBC patients indicated seven major areas of concern: delay in diagnosis, shock, stigma, body image, causal factors and lack of informational and emotional support [58]. Focus group discussions have suggested a particular need for gender-specific information on MBC together with provision of support [59].

In a national study, Brain *et al* administered questionnaires to 161 MBC patients [60]. There was a low incidence of clinical levels of anxiety and depression, 6% and 1%, respectively, but 23% stated that they had cancer-associated distress. These low levels of psychological morbidity may have related to the self-selected nature of the participants. In a much smaller study, Donovan *et al* [61] reported four themes of experience for MBC patients, making sense of the diagnosis, challenge to masculinity, stigma of the diagnosis and the problems of interacting with health-care services. There is an evident need for national protocols for both information and support for men diagnosed with breast cancer.

## Figures and Tables

**Table 1:**
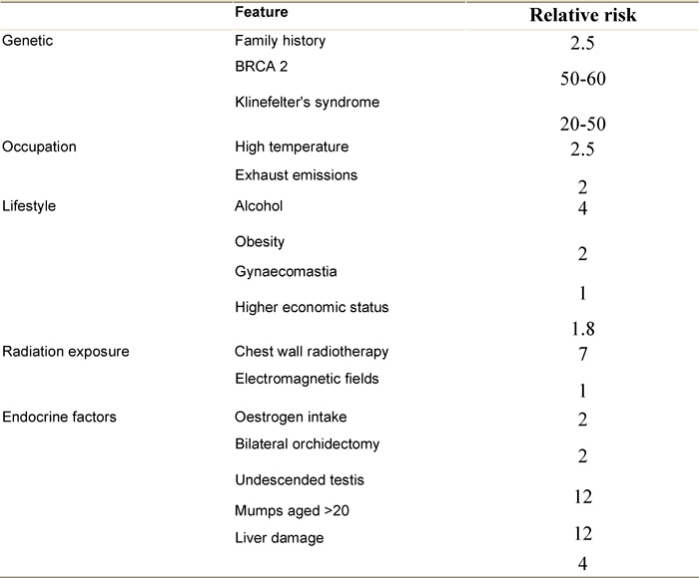
Risk factors for male breast cancer

**Table 2:**
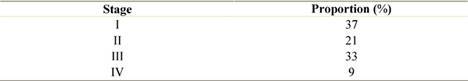
TNM stage at presentation for male breast cancer

**Table 3:**
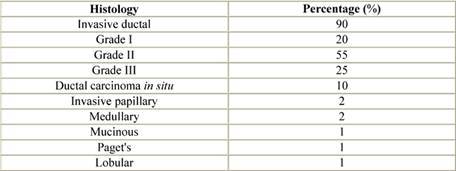
Histopathological types of male breast cancer
